# Sudden unexpected death related to enterovirus myocarditis: histopathology, immunohistochemistry and molecular pathology diagnosis at post-mortem

**DOI:** 10.1186/1471-2334-12-212

**Published:** 2012-09-11

**Authors:** Imed Gaaloul, Samira Riabi, Rafik Harrath, Mark Evans, Nidhal H Salem, Souheil Mlayeh, Sally Huber, Mahjoub Aouni

**Affiliations:** 1 Laboratory of Transmissible Diseases LR99-ES27, Faculty of Pharmacy, Avenue Avicenne, 5000, Monastir, Tunisia; 2 University of Vermont, Department of Pathology, Division of Experimental Pathology, Burlington, USA; 3 Department of Forensic Medicine, University Hospital Fattouma Bourguiba, Monastir, Tunisia. Avenue 1st June, 5000, Monastir, Tunisia; 4 Department of Forensic Medicine, University Hospital Farhat Hached, Sousse, Tunisia. Avenue, Ibn El Jazzar, 4000, Sousse, Tunisia; 5 University of Vermont, Department of Pathology, 208 South Park Drive, Suite #2, Colchester, Vermont, 05446, USA

## Abstract

**Background:**

Viral myocarditis is a major cause of sudden unexpected death in children and young adults. Until recently, coxsackievirus B3 (CVB3) has been the most commonly implicated virus in myocarditis. At present, no standard diagnosis is generally accepted due to the insensitivity of traditional diagnostic tests. This has prompted health professionals to seek new diagnostic approaches, which resulted in the emergence of new molecular pathological tests and a more detailed immunohistochemical and histopathological analysis. When supplemented with immunohistochemistry and molecular pathology, conventional histopathology may provide important clues regarding myocarditis underlying etiology.

**Methods:**

This study is based on post-mortem samples from sudden unexpected death victims and controls who were investigated prospectively. Immunohistochemical investigations for the detection of the enteroviral capsid protein VP1 and the characterization and quantification of myocardial inflammatory reactions as well as molecular pathological methods for enteroviral genome detection were performed.

**Results:**

Overall, 48 sudden unexpected death victims were enrolled. As for controls, 37 cases of unnatural traffic accident victims were studied. Enterovirus was detected in 6 sudden unexpected death cases (12.5 %). The control samples were completely enterovirus negative. Furthermore, the enteroviral capsid protein VP1 in the myocardium was detected in enterovirus-positive cases revealed by means of reverse transcriptase-polymerase chain reaction (RT-PCR). Unlike control samples, immunohistochemical investigations showed a significant presence of T and B lymphocytes in sudden unexpected death victims.

**Conclusions:**

Our findings demonstrate clearly a higher prevalence of viral myocarditis in cases of sudden unexpected death compared to control subjects, suggesting that coxsackie B enterovirus may contribute to myocarditis pathogenesis significantly.

## Background

From a forensic point of view, sudden death is mainly defined as rapid, unexpected and natural death [1]. Most often, sudden unexpected death due to natural causes results from previously unknown cardiovascular diseases though extra cardiac causes should not be ruled out [1,2]. Viral myocarditis, defined clinically as an inflammation of the heart muscle caused by viral infection, is an insidious disease and a major cause of sudden unexpected death [1-6] accounting for approximately 20% of cases in adults under 40. More than 20 viruses have been associated with myocarditis, causing mild to severe injury in the myocardium with ultimate manifestation of end-stage dilated cardiomyopathy and heart failure. Among them, CVB3, a small nonenveloped single-stranded and positive-sense RNA genome of the *Picornaviridae* family and *Enterovirus* genus [7], has been implicated in 25% to 40% of acute myocarditis and dilated cardiomyopathy cases in infants and young adolescents. Myocardial inflammation could be detected in 1 to 9% of routine post-mortem examinations [6]. The clinical diagnosis of myocarditis is made difficult by its variable and nonspecific presentations [4,8-10]. Histopathology is the cornerstone of the diagnosis. A uniform histopathological definition for diagnosis was presented in a consensus statement in 1987 [4,11-13]. According to Dallas criteria, the histopathological diagnosis of myocarditis is based on necrosis or degeneration of the myocytes (or both) and an adjacent inflammatory infiltrate [11,12]. However, the histopathological diagnosis of the disorder remains difficult [14,15]. The relations between the clinical presentation and the histopathological evidence of myocarditis are to be determined. Fatal cases provide a unique opportunity to study these questions as both clinical and histopathological data are available. Furthermore, myocarditis may be falsely recorded as the cause of death if no other obvious causes have been found. In this framework, the current study is aimed at investigating into how accurately viral myocarditis is involved in death when conventional histopathology is supplemented with immunohistochemistry and molecular pathology.

## Methods

### Post-mortem samples

In this study, post-mortem myocardial samples were collected from November 2006 to November 2010. The study included 48 sudden unexpected death cases with clinically suspected inflammatory heart disease. All victims were 18 to 42-year-old males (mean age 29.8 years). The victims’ families denied any previous health problems or coronary artery disease risk factors. The patients were not on any medications and did not give a history of allergy, alcohol or tobacco consumption. The autopsies were performed at two university hospitals located on the coast of Tunisia (Monastir and Sousse). For each sudden unexpected death victim, two myocardial samples (1 cm^3^) were taken from each lobe of the right ventricle, the septum and the left ventricle. The post-mortem myocardial samples were divided into two categories: one fixed in formalin (neutral buffered formaldehyde 30% diluted to 1/10) and embedded in paraffin for histopathology and immunohistochemistry and one frozen and stored at -80°C for RT-PCR. The samples were obtained at autopsy (maximal delay of 12 hours after death). As for controls, post-mortem myocardial samples were collected from 37 cases of unnatural traffic accident deaths. They were 23 to 35 years old (mean age 28.7 years) without any known cardiac pathologies (Table 1).

**Table 1:** Epidemiological data of investigated groups

	**SUD victims (n = 48)**	**Control group (n = 37)**
Age (year)	18-42	23-35
Gender	Male	Male
Cause of sudden death	Clinically suspected inflammatory heart disease	Unnatural traffic accident casualties

The research protocol related to the current study was referred to the Ethics and Medical Research Committees of Farhat Hached University Hospital (Sousse) and Fattouma Bourguiba University Hospital (Monastir). Both committees gave their approval. As for the samples, they were taken in compliance with the Tunisian law (Act 91-22; March 25th 1991) pertaining to human organ removal and transplantation. An individual who has reached the age of majority and who is mentally competent may consent to donate his or her organs for therapeutic or scientific purposes. Thus, an organ may be removed from a cadaver for the above mentioned purposes if the deceased individual had not made known while alive his or her refusal to donate and if this procedure was not opposed by any of the people of full legal capacity (children, parents, spouse, siblings, legal guardian). Accordingly, all samples were taken with the informed consent of the deceased individuals’ next of kin.

### Histopathology: hematoxylin–eosin staining

The post-mortem myocardial samples taken from each sudden unexpected death victim were fixed in formalin (neutral buffered formaldehyde 30% diluted to 1/10) for 24 hours and embedded in paraffin. The sections (5 μm) were cut from the paraffin-embedded tissues with a microtome. All sections were stained with hematoxylin-eosin staining (Invitrogen: Vermont, USA) and the slides were investigated for myocarditis [16-19].

### Immunohistochemical analysis

In all cases, immunohistochemical investigations were performed for CD3-T-lymphocytes (DAKO: Vermont, USA) and CD19-B-lymphocytes (DAKO: Vermont, USA) where the average of counted cells in 20 high power fields (hpf; 400x) was noted. The same investigations were conducted for the enteroviral capsid protein VP1 Mab 5-D8/1 (DAKO: Vermont, USA). Tris-buffered NaCl solution with tween 20, the target retrieval solution, serum-free protein block, antibody diluent, mayer's hematoxylin, EnVision^+^ system-HRP (AEC) and glycergel® mounting medium aqueous were all purchased from DAKO (Vermont, USA). The Immunohistochemical procedures included antigen exposure, blocking, incubation with primary antibody, incubation with the secondary antibody in the En-Vision detection system, and appropriate wash between steps using Tris-buffered saline with tween 20. All incubations were performed at room temperature [20-23]. Briefly, paraffin-embedded tissue sections (5 μm) were dewaxed with xylene and rehydrated with graded ethanol. Antigen exposure was achieved by heat in a water bath (95-99°C) mediated by target retrieval. The endogenous peroxidase activity was blocked with a peroxidase-blocking reagent for 15 minutes. The tissue sections were blocked with the protein block for 10 minutes and then incubated with primary antibody (appropriately diluted to 1:100-1:500 in antibody diluent) for 30 minutes and washed. The secondary antibody in the En-Vision detection system is the goat anti-mouse Ig conjugated with dextran polymer, on which many peroxidase molecules were labeled. The sections were incubated with this reagent for 30 minutes, washed, and then reacted with substrate chromogen for 5-10 minutes. The slides were immersed in aqueous hematoxylin for counterstaining. The color reaction was stopped by a wash in distilled water. Finally, the mounted sections were examined and confirmed under a Nikon Eclipse 50i microscope.

### Reverse transcriptase-polymerase chain reaction (RT-PCR)

RNA was extracted from frozen myocardial tissues using the TRIzol®Plus RNA Purification Kit (Invitrogen: Vermont, USA) according to the suppliers’ protocol. DNase treatment during RNA purification was conducted using PureLink™ DNase (Invitrogen: Vermont, USA) in order to obtain DNA-free total RNA. For the detection of enterovirus RNA, One-step RT-PCR was performed with primers directed to the conserved sequences in the 5'-noncoding region of the enterovirus genome. To confirm that the extracted RNA contained enterovirus RNA, a 155 bp gene fragment was amplified with a one-step RT-PCR (Invitrogen SuperScript™ One-Step RT-PCR with Platinum® Taq: Vermont, USA) using 5 μl of extracted viral RNA and two 5'-noncoding region specific primers 006 and 007 [24]. The RT-PCR was performed with 25 μl 2X reaction mix (a buffer containing 0.4 mM of each dNTP, 2.4 mM MgSO4), 0.2 μM each of sense and anti-sense primers, 1 μl enzyme mix (RT/Platinum® Taq; invitrogen: Vermont, USA), and RNase free water up to 50 μl. The reaction was carried out with an initial reverse transcription step at 42°C for 30 minutes followed by PCR activation at 94°C for 5 minutes, 30 amplification cycles (94°C, 30 seconds; 42°C, 1 minute; 72°C, 2 minutes), and a final 10-minute extension at 72°C in an Eppendorf Mastercycler Thermal Cycler. The PCR-products were run on a 2% agarose gel stained with ethidium bromide and visualized under UV-light.

### Sequencing and analysis of PCR enterovirus amplification products

The PCR amplicons were purified using the ExoSAP-IT – PCR Clean-Up Reagent (USB® Products from Affymetrix, Inc: Vermont, USA) which represents a one-step enzymatic cleanup of PCR products. Then, they were sequenced in forward and reverse directions with the respective PCR primers. The chromatogram sequencing files were inspected with FinchTV (version 1.4.0). The obtained enterovirus sequences were compared to the corresponding ones available in GenBank using the Basic Local Alignment Search Tool in order to identify the enterovirus type [25,26].

## Results

### Histopathological investigation for the detection of inflammatory markers and necrosis factors in the myocardium of sudden unexpected death victims

**Figure 1:**
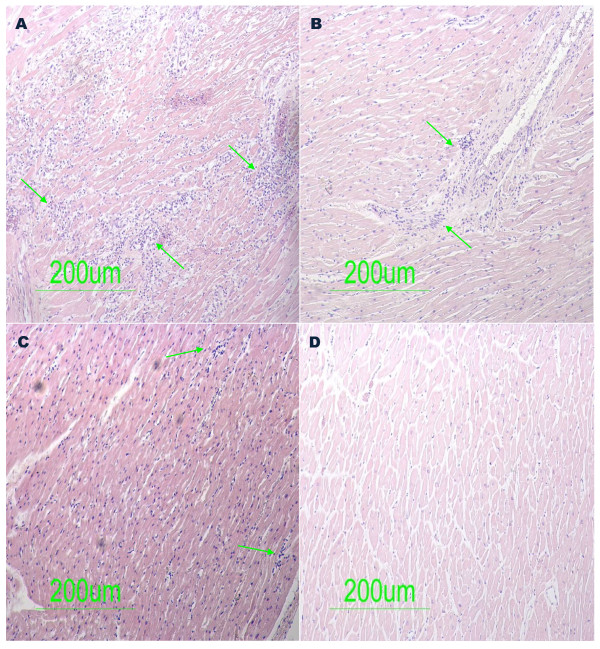
**Histological specimen (hematoxylin-eosin staining) from Sudden Unexpected Death victims demonstrating active myocarditis.** [**1A**, **1B**, **1C**] Areas of diffuse myocardial necrosis with large inflammatory infiltrates (arrows). [**1D**]Control samples (unnatural traffic accident deaths) showing no significant pathological findings.

Having investigated all the paraffin tissue blocks from 48 sudden unexpected death victims with inflammatory cardiovascular disease suspected clinically, active myocarditis was present in four of the 48 cases. Routine histopathological examination revealed these four cases had diffuse inflammation (Figure 1A, B, and C). The slides from the paraffin tissue blocks from 37 cases of unnatural traffic accident victims (control group) showed no significant pathological findings (Figure 1D).

### Immunohistochemical detection of enteroviral capsid protein VP1, CD3-T-lymphocytes and CD19-B-lymphocytes in the myocardium of sudden unexpected death victims

**Figure 2:**
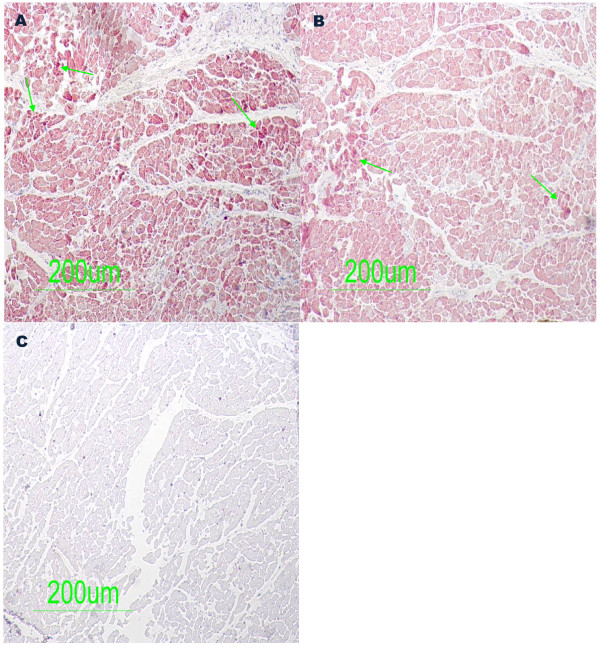
**Immunohistochemical detection of enteroviral capsid protein VP1 in post-mortem myocardial samples.** [**2A**, **2B**] Enteroviral capsid protein VP1 detected inside myocytes (arrows) suggesting a confluent invasion of enterovirus. [**2C**] Control samples (unnatural traffic accident victims) showing no significant pathological findings.

**Figure 3:**
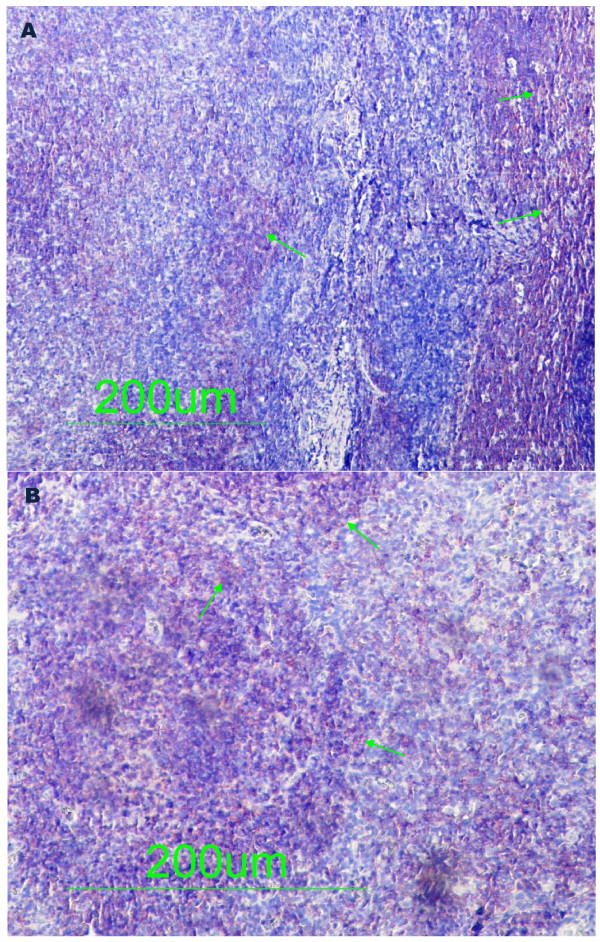
**Immunohistochemical studies for the identification of infected immune cells in the myocardium of sudden unexpected death victims.** [**3A**] Immunohistochemical labeling of paraffin-embedded tissue sections with antibody recognizing T cell (arrows). [**3B**] Immunohistochemical labeling of paraffin-embedded tissue sections with antibody recognizing B cell (arrows).

To determine whether the enterovirus cardiac infection was associated with a viral protein synthesis activity and the presence of infiltrates, all the paraffin tissue blocks taken from sudden unexpected death victims and controls were examined by immunohistochemical assays specific for the enteroviral capsid protein VP1, CD3-T-lymphocytes and CD19-B-lymphocytes. The VP1 capsid protein within the myocardium was detected for all the three tested samples in six of the 48 sudden unexpected death victims (Figure 2A, and B). The slides from the control subjects showed a negative result with no significant pathological findings (Figure 2C). As shown in figure 2 illustrating typical results obtained from six sudden unexpected death victims, the enteroviral capsid protein VP1 appeared to be distributed confluently. The results obtained by quantification of CD3-T-lymphocytes and CD19-B-lymphocytes are listed in Table 2. Unlike control samples, the immunohistochemical investigations revealed a significant presence of CD3-T-lymphocytes and CD19-B-lymphocytes in sudden unexpected death victims having a diffuse distribution (Figure 3A and B).

**Table 2:** Immunohistochemical and molecular-pathological investigations of the myocardium of sudden unexpected death victims

**No**	**Enteroviral capsid protein VP1**	**T-lymphocytes (CD3)**	**B-lymphocytes (CD19)**	**Coxsackie B enterovirus by RT-PCR**
1	+	> 30	> 30	+ (CVB3)
2	+	> 30	27.6	+ (CVB3)
3	+	23.3	> 30	+ (CVB3)
4	+	24.9	> 30	+ (CVB3)
5	+	> 30	> 30	+ (CVB1)
6	+	> 30	> 30	+ (CVB3)

Mean values of T and B-lymphocytes; 20 counted high power fields (HPF; x400); and detection of enteroviral capsid protein VP1.

### Molecular identification of the enterovirus RNA by RT-PCR and sequencing of PCR enterovirus amplification products

**Figure 4:**
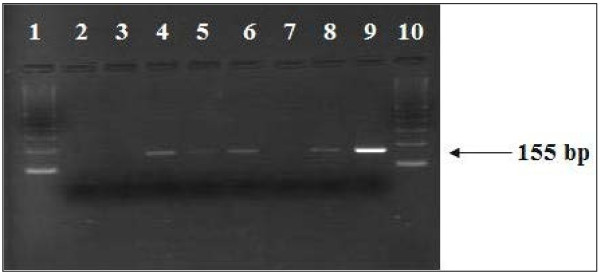
Detection of Coxsackie B enterovirus RNA by RT-PCR in post-mortem myocardial samples from Sudden Unexpected Death victims (1 and 10: molecular size marker 100-bp DNA ladder; 2: Negative control RNA extraction; 3: Negative control RT-PCR mixture; 4 to 6 and 8: Samples from coxsackie B enterovirus-positive cases; 7: Samples from a coxsackie B enterovirus-negative case; 9: A positive control; coxsackievirus B3: 155 bp).

In parallel, the post-mortem myocardial samples were tested for the presence of RNA by RT-PCR using the primers located in the 5'-noncoding region of the enterovirus genome. This marker was positive in at least one of the three samples in six of the 48 tested sudden unexpected death victims. The control samples were negative for enterovirus. Fragments of 155 bp were seen on agarose gel (Figure 4). The sequence analysis of enterovirus-specific RT-PCR assays using RNA extracted from frozen myocardial tissues confirmed the presence of CVB3 in five cases and CVB1 in one.

## Discussion

In a cohort of Tunisian sudden unexpected death victims with inflammatory cardiovascular diseases suspected clinically, the enterovirus from both frozen and formalin-fixed paraffin-embedded post-mortem myocardial samples was identified in 12.5% of cases using histopathology supplemented with immunohistochemistry and molecular-pathology. These findings could have a marked impact on the quality of future investigations in such cases. More importantly, the present results and the investigation methods suggested herein would enable pathologists to better understand and elucidate the cause of death. In this study, the positive results were confirmed using a variety of techniques. Frequency and identification of infectious agents in myocarditis cases have varied widely from 10% to 100% [10,27,28]. These significant differences stem from several factors including the studied sample (serum, cardiac puncture, frozen heart tissue, formalin-fixed paraffin-embedded myocardial samples), the methods used to detect the infectious agent, result confirmation, correlation with clinical and histopathological findings, and the time of illness when the sample was collected.

Enterovirus represents the most common agent of myocarditis. Viral proteins are synthesized as polyprotein, subsequently cleaved to produce the four capsid proteins VP1 to VP4. RT-PCR as well as other techniques revealed an association between enterovirus infection, in particular the cardiotropic coxsackie B enterovirus, serotypes B1–B5, and myocarditis [29]. As an enterovirus group-specific monoclonal antibody (Mab) is now available, the demonstration of enteroviral antigens in myocardium is possible [30]. In the current study, this Mab was used to detect enteroviral antigens in post-mortem myocardial formalin-fixed, paraffin-embedded sections. To demonstrate the expression of CD3-T-lymphocytes and CD19-B-lymphocytes simultaneously as inflammatory markers, immunostaining was performed. In sudden unexpected death victims, the most remarkable finding was the significant presence of those infiltrates consisting of T and B lymphocytes as compared to the control group samples.

With regard to the time-dependent course of viral myocarditis, as studied in the mouse model, early virus-induced myocardial damage already takes place before the histopathological signs of myocarditis defined by the Dallas criteria can be observed [10,16]. Myocarditis misdiagnosis in cases of sudden unexpected death can be overcome with a comprehensive combination of molecular pathological assays and immunohistochemical techniques. This has been confirmed by our findings. All the three samples in six of the 48 tested sudden unexpected death victims were stained positive for the enteroviral capsid protein VP1 and were associated with a significant presence of T and B lymphocytes. More intriguing, they all tested positive for enterovirus RNA by RT-PCR. However, a higher prevalence of viral myocarditis can be revealed by the application of a comprehensive combination of molecular pathological analysis for the detection of enterovirus RNA from frozen heart tissues, and immunohistochemical techniques for the detection of the enteroviral capsid protein VP1 and both CD3-T-lymphocytes and CD19-B-lymphocytes from formalin-fixed, paraffin-embedded heart tissue. This is of great importance when a routine histopathological examination alone does not yield reliable findings in heart muscle tissue.

## Conclusion

In conclusion, the present study based on sudden unexpected death cases on the coast of Tunisia demonstrates clearly a higher prevalence of viral myocarditis suggesting that coxsackie B enterovirus may contribute to myocarditis pathogenesis significantly. The combined investigations using histopathology, molecularpathological techniques and immunohistochemical methods were able to reveal the causative agents of death. Prospective murin experimental myocarditis would be desirable and could have a marked impact on the quality of future investigations to better understand and elucidate the physio and immuno-pathogenesis of such infectious heart diseases.

## Competing interests

The authors have no commercial disclosures to make with regard to this manuscript. No competing financial interests exist.

## Authors’ contributions

IG initiated and designed the study and was in charge of specimens and clinical information collection as well as data analysis, histopathological, molecular and immunohistochemical studies and preparation of the manuscript. SR carried out the analysis and interpretation of data and prepared the manuscript. RH helped draft the manuscript and was involved in revising the manuscript critically for intellectual content. ME participated in the design of the methodology. NHS Helped in the collection of samples from the Department of Forensic Medicine at the University Hospital Fattouma Bourguiba, Monastir. SM Helped in the collection of samples from the Department of Forensic Medicine at the University Hospital Farhat Hached, Sousse. SH initiated the study and participated in its design and coordination. MA initiated the study, and participated in its design and coordination. All authors read and approved the final manuscript.

## Pre-publication history

The pre-publication history for this paper can be accessed here:

http://www.biomedcentral.com/1471-2334/12/212/prepub
